# Supplementary figures and images for: Stochastic modeling of a gene regulatory network driving B cell development in germinal centers
Source: PLoS One. 2024 Mar 28;19(3):e0301022. doi: 10.1371/journal.pone.0301022 (PMC10977792; doi:10.1371/journal.pone.0301022)

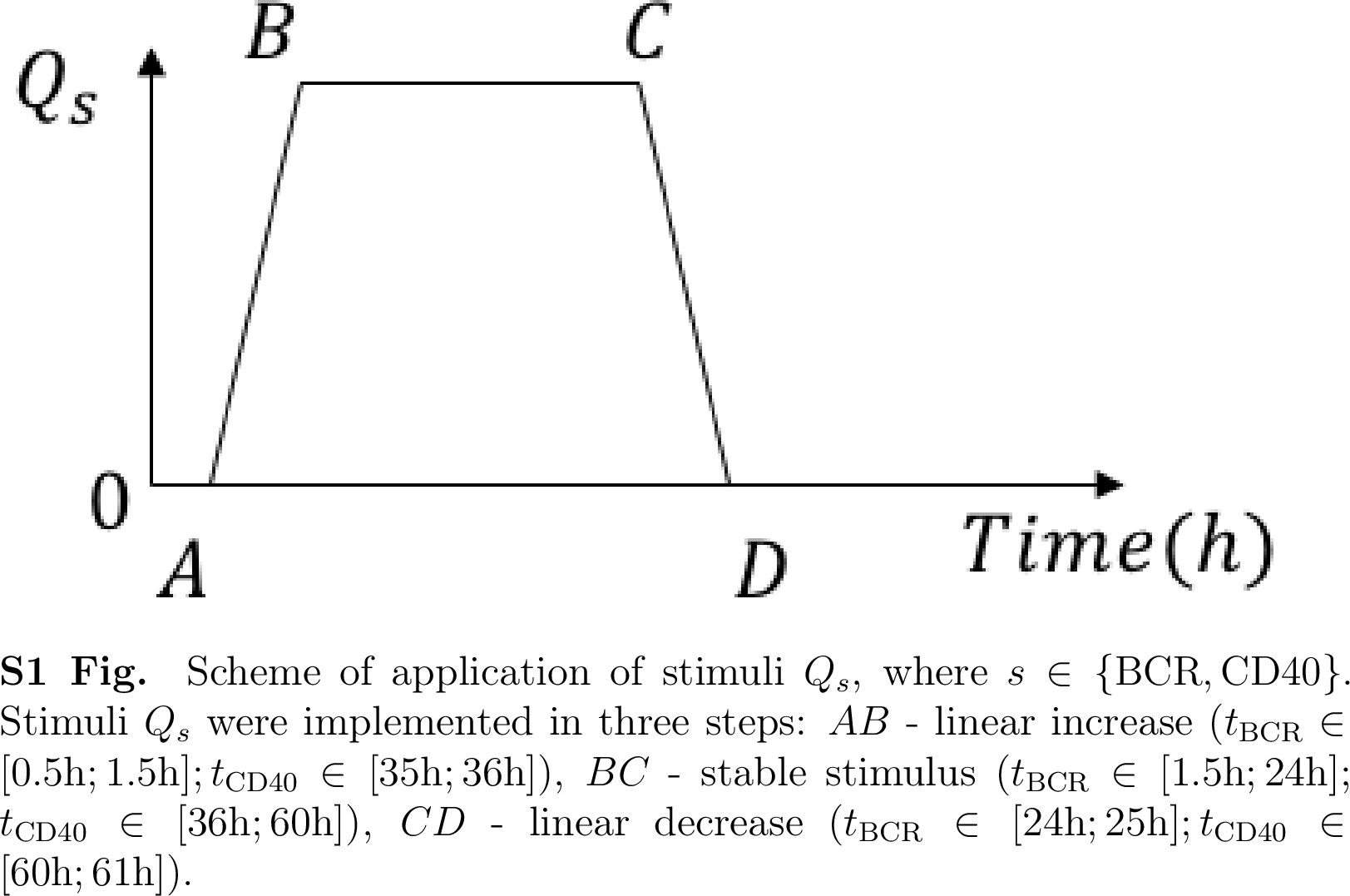

Supplement: S1 Fig — (TIF) [file pone.0301022.s001.tif]

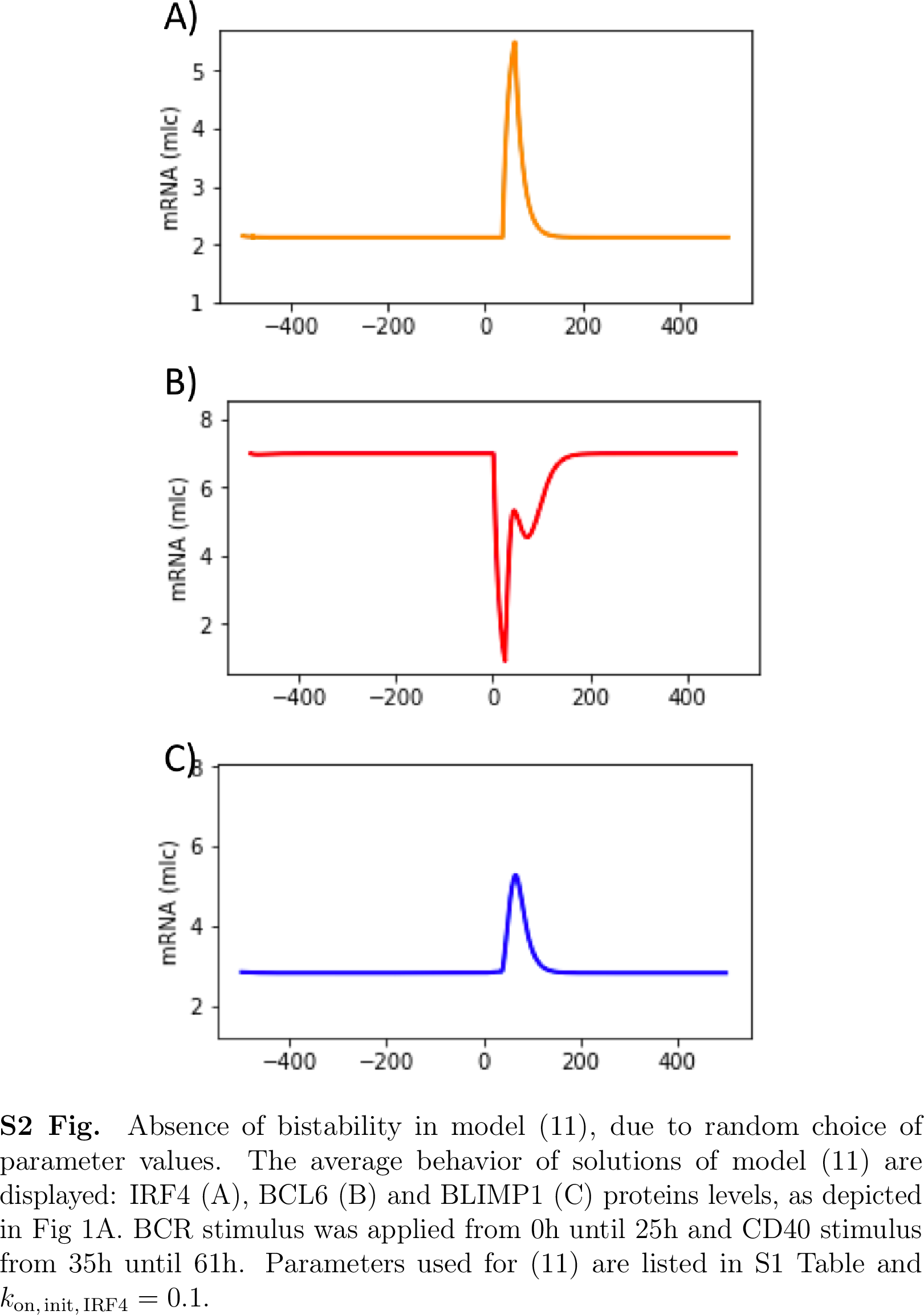

Supplement: S2 Fig — (TIF) [file pone.0301022.s002.tif]

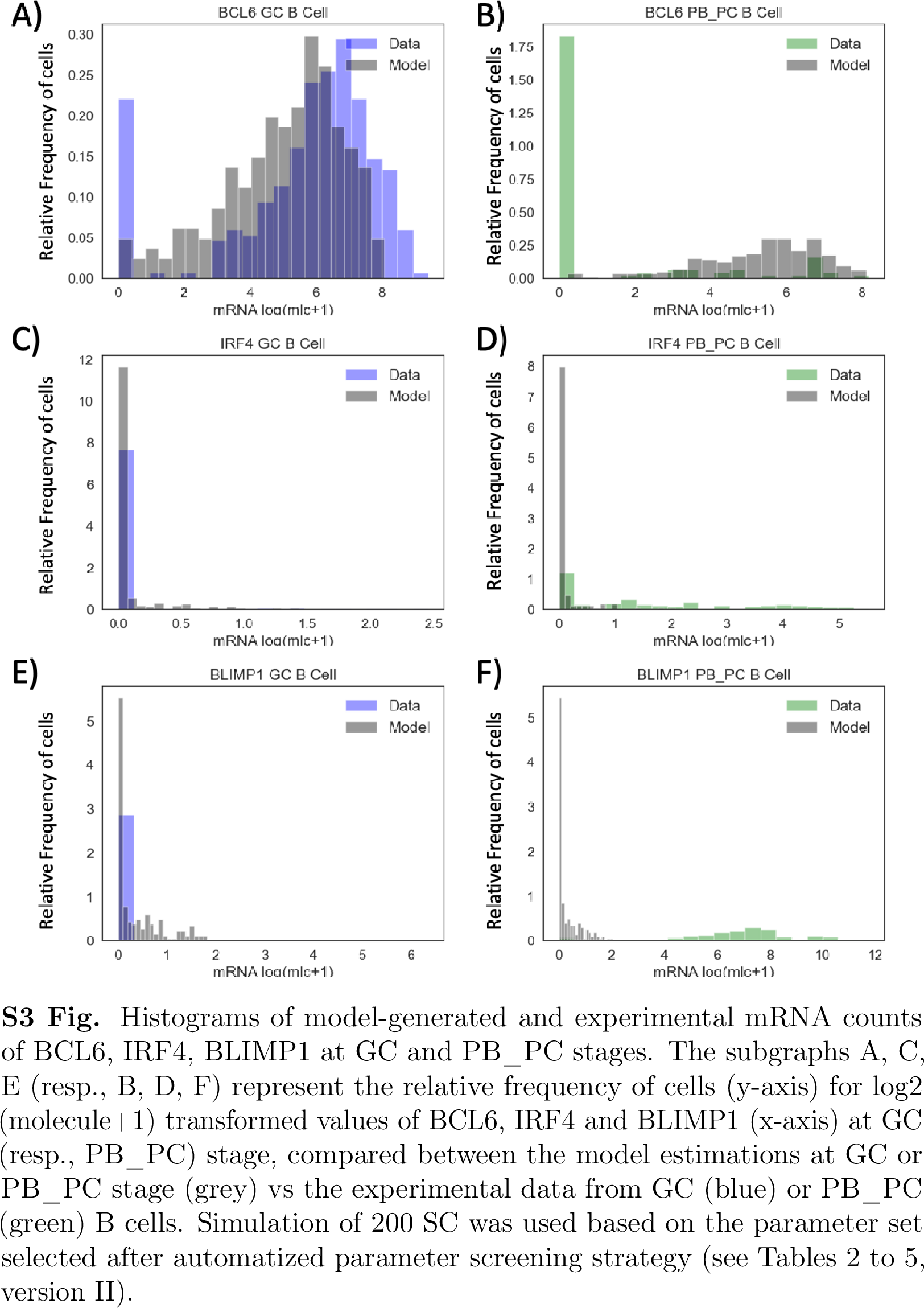

Supplement: S3 Fig — (TIF) [file pone.0301022.s003.tif]
