# Supplementary material for: Stochastic modeling of a gene regulatory network driving B cell development in germinal centers
Source: PLoS One. 2024 Mar 28;19(3):e0301022. doi: 10.1371/journal.pone.0301022 (PMC10977792; doi:10.1371/journal.pone.0301022)
Supplement: S1 File — This file introduces the methodology for reducing the stochastic model and estimating the initial activation rate. (PDF) [file pone.0301022.s004.pdf]

SI File 1: Modeling

Stochastic modeling of a gene regulatory network driving B cell development in germinal centers

A. Koshkin, U. Herbach, M. Rodríguez Martínez, O. Gandrillon, F. Crauste

This file introduces the methodology for reducing the stochastic model and estimating the initial activation rate. Equations from the main manuscript are labeled (X) while equation in this supporting file are labeled (SI.X).

From Martinez et al. [1], one can see that IRF4 is a crucial node and its autoactivation reaction is responsible for the bistability switch from the GC to PB_PC during B cell differentiation. Notably, equation (3) which describes the dynamics of IRF4, only depends on IRF4. Based on these observations, we have decided to use IRF4 as a connecting edge between models (4)-(6) and (1)-(3).

Starting from the stochastic model (4)-(6) written for IRF4, we reduced it to an ODE model by making a simplifying assumption. We substitute the stochastic process $E(t)$ by its mean value $\langle E(t) \rangle$, so

$$\begin{cases} \dfrac{dM_{\text{IRF4}}}{dt} &= s_{0,\,\text{IRF4}} \langle E_{\text{IRF4}}(t) \rangle - d_{0,\,\text{IRF4}} M_{\text{IRF4}}(t), \\ \dfrac{dP_{\text{IRF4}}}{dt} &= s_{1,\,\text{IRF4}} M_{\text{IRF4}}(t) - d_{1,\,\text{IRF4}} P_{\text{IRF4}}(t). \end{cases} \tag{SI.1}$$

We are looking for parameter values of system (SI.1) that will allow to reproduce the same behavior than the kinetic model (1)-(3) (i.e. two steady states). At steady state, system (SI.1) with $\frac{dM}{dt} = 0$ and $\frac{dP}{dt} = 0$ leads to:

$$P_{\text{IRF4}}(t) = \frac{s_{1,\,\text{IRF4}} s_{0,\,\text{IRF4}} \langle E_{\text{IRF4}}(t) \rangle}{d_{0,\,\text{IRF4}} d_{1,\,\text{IRF4}}}. \tag{SI.2}$$

Introducing the new variable

$$c = \frac{s_{1,\text{ IRF4}} s_{0,\text{ IRF4}}}{d_{1,\text{ IRF4}} d_{0,\text{ IRF4}}}$$

we can write (SI.2) as

$$P_{\text{IRF4}}(t) = c\langle E_{\text{IRF4}}(t)\rangle. \tag{SI.3}$$

In Martinez et al. [1], IRF4 behavior was described by equation (3), that is

$$\frac{dr}{dt} = \mu_r + \sigma_r \frac{r^2}{k_r^2 + r^2} + CD40 - \lambda_r r,$$

i.e. with our notation it can be written as:

$$\frac{dp_{\text{IRF4}}}{dt} = \mu_{\text{IRF4}} + \sigma_{\text{IRF4}} \frac{p_{\text{IRF4}}^2}{k_{\text{IRF4}}^2 + p_{\text{IRF4}}^2} + CD40 - \lambda_{\text{IRF4}}\, p_{\text{IRF4}}. \tag{SI.4}$$

Assuming that CD40 = 0 at the beginning of the simulation, that equation (SI.4) is at the steady state, that λ_{IRF4} is a degradation rate of protein for IRF4 and using (SI.3), we write (SI.4) as:

$$\mu_{\text{IRF4}} + \sigma_{\text{IRF4}} \frac{c^2(\langle E_{\text{IRF4}}(t)\rangle)^2}{k_{\text{IRF4}}^2 + c^2(\langle E_{\text{IRF4}}(t)\rangle)^2} - c d_{1,\text{ IRF4}} \langle E_{\text{IRF4}}(t)\rangle = 0. \tag{SI.5}$$

Solving equation (SI.5) in terms of $\langle E_{\text{IRF4}}(t)\rangle$ leads to

$$\begin{aligned} 0 = {} & c^3 d_{1,\text{ IRF4}}(\langle E_{\text{IRF4}}(t)\rangle)^3 - (\mu_{\text{IRF4}} + \sigma_{\text{IRF4}})c^2(\langle E_{\text{IRF4}}(t)\rangle)^2 \\ & + c d_{1,\text{ IRF4}} k_{\text{IRF4}}^2(\langle E_{\text{IRF4}}(t)\rangle) - \mu_{\text{IRF4}} k_{\text{IRF4}}^2 \end{aligned}$$

and can be simplified in the form:

$$a'(\langle E_{\text{IRF4}}(t)\rangle)^3 - b'(\langle E_{\text{IRF4}}(t)\rangle)^2 + c'(\langle E_{\text{IRF4}}(t)\rangle) + d' = 0 \tag{SI.6}$$

where

$$\begin{cases} a' = c^3 d_{1,\text{ IRF4}} \\ b' = (\mu_{\text{IRF4}} + \sigma_{\text{IRF4}})c^2 \\ c' = c d_{1,\text{ IRF4}} k_{\text{IRF4}}^2 \\ d' = -\mu_{\text{IRF4}} k_{\text{IRF4}}^2 \end{cases}$$

Because parameters a', b', c' are positive and d' is negative, there is at least one positive root to (SI.6). Further, we fitted the parameters

$\mu_{\mathrm{IRF4}}, \sigma_{\mathrm{IRF4}}, k_{\mathrm{IRF4}}$, applying fitting procedure from Martinez et al. [1] and using the experimental data accession no. GSE 12195 (see Tables 2 tot 5), and we found that $E_{\mathrm{IRF4}}(t_{\mathrm{init}})$ value which would correspond to a bistable regime of system (1)-(3) is:

$$E_{\mathrm{IRF4}}(t_{\mathrm{init}}) = 1.7 \times 10^{-3}.$$

We also know that

$$\langle E_{\mathrm{IRF4}} \rangle = \frac{k_{\mathrm{on}}}{k_{\mathrm{on}} + k_{\mathrm{off}}},$$

so assuming that at initial time $t = t_{\mathrm{init}}$, $k_{\mathrm{on}} << k_{\mathrm{off}}$ and $k_{\mathrm{on}} = \alpha k_{\mathrm{off}}$, with $\alpha << 1$, one can define $\alpha = E_{\mathrm{IRF4}}/(1 - E_{\mathrm{IRF4}}) = 1.7 \times 10^{-3}$. Assuming $k_{\mathrm{off}} \approx 1$ ($k_{\mathrm{off,\,init,\,IRF4}} \approx 1$) allows to estimate the value of k_{on} for IRF4, which should keep reduced model (11) in a two steady state regime:

$$k_{\mathrm{on,\,IRF4}} = 1.7 \times 10^{-3} \tag{SI.7}$$

Further, we called the value (SI.7), the initial value $k_{\mathrm{on,\,init}}$ for IRF4.

References

[1] María Rodríguez Martínez, Alberto Corradin, Ulf Klein, Mariano Javier Álvarez,Gianna M Toffolo, Barbara di Camillo, Andrea Califano, and Gustavo A Stolovitzky.“Quantitative modeling of the terminal differentiation of B cells and mechanismsof lymphomagenesis”. In:Proceedings of the National Academy of Sciences109.7(2012), pp. 2672–2677.
