# Supplementary material for: Stochastic modeling of a gene regulatory network driving B cell development in germinal centers
Source: PLoS One. 2024 Mar 28;19(3):e0301022. doi: 10.1371/journal.pone.0301022 (PMC10977792; doi:10.1371/journal.pone.0301022)
Supplement: S1 Table — (PDF) [file pone.0301022.s005.pdf]

Parameter	Values	Parameter	Values
$H_{1,1}$	1	$s_{0,\mathrm{BCL6}}$	6.5
$H_{2,1}$	0.1	$s_{0,\mathrm{IRF4}}$	2
$H_{3,1}$	1	$s_{0,\mathrm{BLIMP1}}$	6.5
$H_{1,2}$	1	$d_{0,\mathrm{BCL6}}$	0.05
$H_{2,2}$	0.01	$d_{0,\mathrm{IRF4}}$	0.05
$H_{3,2}$	1	$d_{0,\mathrm{BLIMP1}}$	0.1733
$H_{1,3}$	0.1	$s_{1,\mathrm{BCL6}}$	100
$H_{2,3}$	0.001	$s_{1,\mathrm{IRF4}}$	160
$H_{3,3}$	1	$s_{1,\mathrm{BLIMP1}}$	40
$H_{\mathrm{BCR},1}$	0.01	$d_{1,\mathrm{BCL6}}$	0.138
$H_{\mathrm{CD40},2}$	1	$d_{1,\mathrm{IRF4}}$	0.173
$\theta_{1,1}$	-0.2	$d_{1,\mathrm{BLIMP1}}$	0.173
$\theta_{2,1}$	-10	$k_{\mathrm{on,\,init,\,BCL6}}$	0.1
$\theta_{3,1}$	-2	$k_{\mathrm{on,\,init,\,IRF4}}$	0.1
$\theta_{1,2}$	0	$k_{\mathrm{on,\,init,\,BLIMP1}}$	0.1
$\theta_{2,2}$	8	$k_{\mathrm{off,\,init,\,BCL6}}$	1
$\theta_{3,2}$	0	$k_{\mathrm{off,\,init,\,IRF4}}$	1
$\theta_{1,3}$	-1	$k_{\mathrm{off,\,init,\,BLIMP1}}$	1
$\theta_{2,3}$	40		
$\theta_{3,3}$	0		
$\theta_{\mathrm{BCR},1}$	-200		
$\theta_{\mathrm{CD40},2}$	10		

S1 Table. Parameters of System (11) with values accordingly to Bonnaffoux et al. [32].
