# Supplementary material for: Stochastic modeling of a gene regulatory network driving B cell development in germinal centers
Source: PLoS One. 2024 Mar 28;19(3):e0301022. doi: 10.1371/journal.pone.0301022 (PMC10977792; doi:10.1371/journal.pone.0301022)
Supplement: Attachment — Submitted filename: Response-to-reviewers.pdf [file pone.0301022.s006.pdf]

Response to Reviewers' comments on the manuscript entitled "Stochastic modeling of a gene regulatory network driving B cell development in germinal centers", by Koshkin et al.

We want to thank the Editor and the reviewers for their comments and suggestions. We tried to answer all questions and comments. Our answers below mention modifications made to the manuscript by indicating pages and lines. Modifications are highlighted in the manuscript, as required by the Journal guidelines.

Reviewer #1.

The manuscript by Alexey Koshkin et al. deals with the investigation of the gene regulatory networks (GRNs) associated with germinal center (GC) cell development and differentiation based on public available single-cell (SC) transcriptomic data. Including three key gene regulators (BCL6, IRF4, BLIMP1), influenced by two external stimuli signals (surface receptors BCR and CD40), a model was established that qualitatively recapitulates mRNA distributions corresponding to GC and plasmablast stages of B cell differentiation, which can be used in validating the GRN in physiological and pathophysiological conditions. The manuscript is written well and meets almost all criterias for publishing in PlosOne.
Comments: The validation (testing) of the model on a test-data set (for example the sc-RNA data set from the same sample source but also an external data set) would highly improve the quality of the paper.

Authors' response: We concur with Reviewer # 1 regarding the potential benefits of testing the robustness of model (4)-(6) using an alternative single cell dataset. Originally, the model developed in this paper was supposed to be calibrated on experimental single cell data generated by our collaborators in the COSMIC project (COSMIC grant from European Union's Horizon 2020 research and innovation program under the Marie Skłodowska-Curie grant agreement no. 765158). Yet, COVID-19 pandemic redirected resources and research time on emergency matters and our international collaboration was not able to deliver expected datasets. Consequently, we opted to use the SC data from existing literature and selected the single cell data introduced by Milpied et al. [2] due to their high quality and relevance. This approach reflects a common practice in the methodological publications, where parameter's fitting is based on the single dataset. Indeed, fitting parameters to a single dataset and using the trained model for in silico experiments is standard in a lot of methodological

papers, which often lack access to perturbational datasets.
At this stage, testing the model's performances would require a new dataset generated by mutating one of the genes (a knock-out of one of the main genes for instance). Indeed, using another dataset obtained in the normal B cell development situation would basically be doing the same thing again, while introducing a mutation would allow to investigate the model's ability to predict mRNA distributions. However, designing, performing and studying such an experiment is beyond the scope of this paper. We do hope this decision will be accepted by the reviewer.

Reviewer #2.

In this paper the authors use stochastic modeling of a gene regulatory network to fit single cell expression data related to B cell differentiation in germinal centers. This is an important problem which can lead to a better understanding of malignancies in B cells. The work is technically sound, and the paper is well-written with the mathematical descriptions and the figures doing a good job of clearly presenting the work to the readers. I would like to point out some revisions that are still needed.

- The benefit of using a stochastic model over a kinetic model, as also the improvements of using Version III over Version II are not fully clear without including a figure like Figure 2 each for the latter two parameter tune cases. For example, in version III PB_PC improves for two genes, however, the GC performance becomes worse. So plots showing the mRNA counts like in Figure 2 would show how close the total model predictions are to the observed values.

Authors' response: First, we want to discuss the reason that motivated us to introduce Figure 2. The goal of reduced system (11) was to identify an initial value of the promoter state $E(t)$ for IRF4 gene such that system (11) would exhibit two steady states for IRF4. Figure 2 highlights the behavior of reduced system (11). However, due to the deterministic nature of the kinetic model, it could not be used to describe single cell data. This is why we developed a probabilistic model (system (4)-(6)).
Second, due both to the nature of single cell data and the probabilistic nature of our model (4)-(6), reducing our results to the ability of the model to fit data corresponding only to average values, plus/minus standard deviations, as shown in Figure 2, would not be in line with the objective of the current work. This is why we showed our model's results in the form of histograms and used the Kantorovith distance (KD, section Evaluation of model variability using

Kantorovich distance, line 189) to evaluate which version of the model better fitted data. This approach is adapted to single cell data, contrary to results similar to the ones in Figure 2 that would not emphasize the richness of single cell data.
We are sorry that the initial submission of our work was not clear enough on these aspects. We have added a sentence to section 3.1 in order to clarify further tuning of the parameters for model (4)-(6). The modification reads (page 10, lines 238-241): "Due to the stochastic nature of gene expression, we are hereafter interested in evaluating how stochastic system (4)-(6) is capable of simulating this stochastic behavior in B cell differentiation in GC and recapitulates the shape of the mRNA distributions from the experimental SC dataset."

- Some of the parameters depicted in equations (4)-(6) are not present later, for example, β_i, γ, k^{min}, k^{max}. It needs to be clarified how they are replaced (e.g. maybe by k_{init}) or not used anymore.

Authors' response: This information should have been available in our submission, we apologize for this mistake. First, we corrected equation (6) and its description (page 4, equation (6) and lines 87-89, 93-94, 98-99) so that all parameters appear clearly in the text and the formula (threshold values $H_{s,i}$ for stimuli Q_s were missing). All parameters mentioned by Reviewer #1 were initially estimated following the approach in [1], and were no longer modified through the study. We added a paragraph in the manuscript (pages 4-5, lines 108-113): "Following the approach in [1], the values of β_i were computed when initializing the simulation, in order to set the values of parameters $k_{\mathrm{on},i}$ and $k_{\mathrm{off},i}$ to their initial values. Parameter γ was set to a default value, equal to 2, and values of $k^{max}_{\mathrm{on},i}$ and $k^{max}_{\mathrm{off},i}$ were estimated by the method of moments and bootstrapping as previously described by Bonnaffoux et al. [1]. These parameters were no longer modified through this study."

- In Pages 6 and 7, line 146-154, the ranges for some parameters are not given, e.g. $\theta_{1,1}$, $H_{1,1}$, etc. In line 152, aren't there 6 $H_{j,i}$ interactions and 11 $\theta_{i,j}$ interactions? The values in Line 153 do not match, so that section needs to be corrected so that the number of parameter combinations adds up.

Authors' response: We have corrected the text in section Estimation of the parameters for the stochastic model: Automatized approach (line 149) and Tables 2 to 6 in order to specify more clearly which parameters were tested and which values were tested for each parameter. There were many wrong parameter values reported in the manuscript, we apologize for these mistakes. Tables 2 to 5 have been corrected when necessary (corrections are highlighted, some values

were not correctly reported), and our modifications of the text appear on pages 6-7-8, lines 155-172:

"We investigated a possible effect of $H_{w,i}$ values ($w = 1, 2, 3$, BCR, CD40), $\theta_{j,i}$ values and $k_{\text{on, init}}$ values on the quality of the fitting (for each parameter combination, simulation was performed for 200 SC).

First, let us mention that, based on the network depicted in Figure 1, there is no influence of BCL6 on IRF4, of BLIMP1 on IRF4 and there is no self-activity of BLIMP1 on itself, so parameters $\theta_{1,2}$, $\theta_{3,2}$ and $\theta_{3,3}$ are set to 0 while parameters $H_{1,2}$, $H_{3,2}$ and $H_{3,3}$ are set to 1. Also, BCR acts only on BCL6 and CD40 on IRF4, so $\theta_{\text{BCR},2} = \theta_{\text{BCR},3} = 0$, $\theta_{\text{CD40},1} = \theta_{\text{CD40},3} = 0$, and only parameters $\theta_{\text{BCR},1}$ and $\theta_{\text{CD40},2}$ are non-zero.

We tested the values of interaction threshold $H_{1,1}$, $H_{1,3}$, $H_{2,1}$, $H_{2,3}$ and $H_{3,1}$ within the set $\{0.01, 0.1, 1\}$, and the set $\{0.0001, 0.001, 0.1, 1, 100\}$ for $H_{2,2}$, $H_{BCR,1}$, $H_{CD40,2}$. We also tested the values of $\theta_{1,1}$, $\theta_{1,3}$, $\theta_{2,1}$, $\theta_{2,3}$ and $\theta_{3,1}$ by multiplying by a factor $f_\theta \in \{1,\ 5\}$, and by multiplying by a factor $f_\theta \in \{1, 10\}$ for $\theta_{2,2}$, $\theta_{\text{BCR},1}$, $\theta_{\text{CD40},2}$.

In total we tested two different values of $\theta_{j,i}$ for 5 interactions ($\theta_{1,1}$, $\theta_{1,3}$, $\theta_{2,1}$, $\theta_{2,3}$, $\theta_{3,1}$), 2 values of $\theta_{j,i}$ for 3 interactions ($\theta_{2,2}$, $\theta_{\text{BCR},1}$, $\theta_{\text{CD40},2}$), 3 values of $H_{j,i}$ for 5 interactions ($H_{1,1}$, $H_{1,3}$, $H_{2,1}$, $H_{2,3}$, $H_{3,1}$), and 5 values of $H_{j,i}$ for 3 interactions ($H_{\text{BCR},1}$, $H_{\text{CD40},2}$, $H_{2,2}$), generating $2^5 \times 2^3 \times 3^5 \times 5^3 \approx 7.8 \times 10^6$ combinations of parameters. Parameters that do not appear in the previous list have not been tested."

- Why is CD40 stimuli up to 61 instead of 60?

Authors' response: This was a mistake, CD40 stimuli are actually applied up to 61h, not 60h as indicated in our initial submission. This has been corrected (page 5, line 126).

- In Figures 4, 5, S3, some further details about the y-axis scale are needed for the reader.

Authors' response: We modified the captions of figures 4, 5 and S3, as well as their y-axis labels.

- In Figure 1 caption, $k_{off,i}$ is not shown to be dependent on P_j and $\theta_{j,i}$ in the text, only $k_{on,i}$, so those should match. Also, the autoactivation loop for BCL6 needs to be mentioned, as it is mentioned just for IRF4.

Authors' response: We modified the caption of Figure 1 to account for the reviewers' comments. We have added that both $k_{\text{on},i}$ and $k_{\text{off},i}$ depend on P_j and

$\theta_{j,i}$, and we have also included the fact that BCL6 has an autorepression loop: "The interaction between a regulator gene j and a target gene i is defined by the dependence of both $k_{\text{on},i}$ and $k_{\text{off},i}$ on the protein level P_j and the interaction parameter $\theta_{j,i}$. IRF4 gene exhibits an autoactivation loop ($\theta_{2,2}$). BCL6 gene exhibits an autorepression loop ($\theta_{1,1}$)."

We also modified the text to explicitly define $k_{\text{off},i}$ (page 4, line 100): "For defining $k_{\text{off},i}$, all $\theta_{i,j}$ values must be replaced by -$\theta_{i,j}$ in (6)"

- Error in Line 363 (antibody producing, not antigen producing) should be corrected

Authors' response: This typo has been corrected (page 13, lines 385-386).

References

[1] Bonnaffoux A, Herbach U, Richard A, Guillemin A, Gonin-Giraud S, Gros P-A, Gandrillon O. (2019) WASABI: a dynamic iterative framework for gene regulatory network inference. BMC Bioinformatics 20(1): 1–19.

[2] Milpied P, Cervera-Marzal I, Mollichella M-L, Tesson B, Brisou G, Traverse-Glehen A, et al. (2018) Human germinal center transcriptional programs are desynchronized in B cell lymphoma. Nat Immunol 19(9): 1013–1024.
